# Informing implementation of quality improvement in Australian primary care

**DOI:** 10.1186/s12913-018-3099-5

**Published:** 2018-04-16

**Authors:** Charlotte Hespe, Lucie Rychetnik, David Peiris, Mark Harris

**Affiliations:** 1 The University of Notre Dame Australia, Level 2, 160 Oxford St Darlinghurst, Sydney, NSW 2010 Australia; 2 The George Institute for Global Health, Level 5, 1 King Street Newtown, Sydney, 2042 Australia; 3 Centre for Primary Health Care and Equity, Level 3 AGSM , UNSW Kensington, Sydney, NSW 2052 Australia

**Keywords:** Quality improvement, Primary care, Implementation, Leadership, Organisational culture, Data, Primary health care organisations

## Abstract

**Background:**

Quality Improvement (QI) initiatives in primary care are effective at improving uptake of evidence based guidelines, but are difficult to implement and sustain. In Australia meso-level health organisations such as Primary health care Organisations (PHCO) offer new opportunities to implement area-wide QI programs. This study sought to identify enablers and barriers to implementation of an existing Australian QI program and to identify strategic directions that PHCOs can use in the ongoing development of QI in this environment.

**Methods:**

Semi-structured telephone interviews were conducted with 15 purposively selected program staff and participants from the Australian Primary Care Collaborative (APCC) QI program. Interviewees included seven people involved in design, administration and implementation of the APCC program and eight primary care providers (seven General Practitioners (GPs) and one practice nurse) who had participated in the program from 2004 to 2014. Interviewees were asked to describe their experience of the program and reflect on what enabled or impeded its implementation. Interviews were recorded, transcribed and iteratively analysed, with early analysis informing subsequent interviews. Identified themes and their implications were reviewed by a GP expert reference group.

**Results:**

Implementation enablers and barriers were grouped into five thematic areas: (1) leadership, particularly the identification and utilisation of change champions; (2) organisational culture that supports quality improvement; (3) funding incentives that support a culture of quality and innovation; (4) access to and use of accurate data; and 5) design and utilisation of clinical systems that enable and support these issues. In all of these areas, the active involvement of an overarching external support organisation was considered a key ingredient to successful implementation.

**Conclusion:**

There are substantial opportunities for PHCOs to play a pivotal role in QI implementation in Australia and internationally. In developing QI programs and policies, such organisations ought to invest their efforts in: (1) identifying and mentoring local leaders; (2) fostering QI culture via development of local peer networks; (3) developing and advocating for alternative funding models to support and incentivise these activities; (4) investing in data and audit tool infrastructure; and (5) facilitation of systems implementation within primary care practices.

**Electronic supplementary material:**

The online version of this article (10.1186/s12913-018-3099-5) contains supplementary material, which is available to authorized users.

## Background

The Australian primary health care system was initially designed to deliver acute care services but has been modified over twenty five years to better address preventive health and chronic disease management. This evolution started in 1992 with the release of “National Health Strategy: the Future of General Practice” [1]. The strategy promoted development of general practice standards, accreditation and integration with health services outside general practice. In 2010 the first national Primary Healthcare strategy was launched identifying a focus on integration, information technology, access, quality, and safety as some of the key building blocks and priority areas [2].

Despite this evolution from reactive to more proactive health care, large gaps are evident in the quality of care received. CareTrack, an Australian study conducted during 2009–2010, reported only 57% of patients with chronic conditions in primary care encounters received appropriate care [3]. Barriers that prevent the adoption of best practice often appear to overwhelm clinicians and managers working in primary health care [4].

Quality Improvement (QI) initiatives in primary care have the potential to improve uptake of evidence based practices, but have been difficult to implement and sustain [5]. QI is a multi-dimensional concept which, in the healthcare context, can be defined as having a systematic approach to making changes that will lead to better patient outcomes (health), better system performance (care) and better professional development (learning) [6]. Bataldan et al. postulate that by defining QI in this way, it allows people to have a measureable approach to the concept of improving healthcare. There are a number of ways to intentionally implement QI initiatives, and one such approach is the establishment of Quality Improvement Collaboratives (QIC). QICs actively bring together groups of practitioners from different organisations to meet and learn about a specific aspect of health service quality and to share experiences about making changes in their local settings. The process specifically supports practitioners to use QI tools such as Plan, Do, Study, Act (PDSA) cycles to achieve improvements.

There has been mixed evidence of success using QICs in health care. A systematic review of 64 QIC programs in 2018 reported significant improvements in 83% of targeted clinical processes and patient outcomes [7]. The authors noted that enthusiasm for these findings must be tempered by reflection on the limitations in design and reporting in many QIC’s, as well as likely publication bias. Evidence suggests that implementation processes for each QIC may be critical drivers of program success with up to 66 contextual factors identified in a 2014 study as associated with improved outcomes [8].

**Fig. 1:**
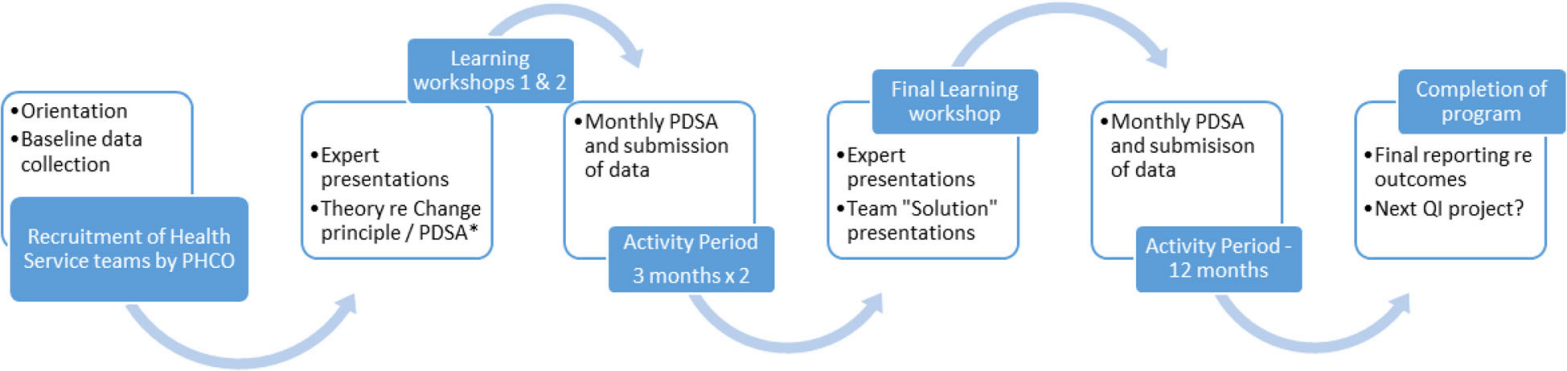
Elements of the APCC program wave

In Australia the Improvement Foundation (IF) has administered a series of QICs called the Australian Primary Care Collaboratives (APCC) since 2004 ([9]). The program was designed in collaboration with the United Kingdom National Primary Care Development Team [10]. The IF was initially commissioned by the Australian Federal government to assist primary care practices in five priority areas; coronary heart disease, diabetes, chronic obstructive pulmonary disease, chronic disease prevention and self-management, and health care access and care redesign. APCC offered General Practices and Aboriginal health services financial incentives to participate in a QIC wave over 18 months. A wave (Fig. 1) comprised an orientation session to the principles of QI, followed by a series of learning workshops (delivered either face-to-face or online). The goal was for each primary care practice to complete requirements of one “wave” during which time Practice staff would each develop a knowledge of QI, understand the principles of measuring for improvement, the use of data in QI, the application of QI tools as well as identify ways to build a team culture and develop effective communication skills to facilitate QI. Clinical and administration staff were expected to attend workshops and submit monthly data to monitor their progress. One thousand one hundred and eighty five primary care health services (16% of all Australian General Practices) and 83% of PHCO’s enrolled in the APCC program between 2004 and 2012 [11]. Practice characteristics varied enormously from solo clinician to large multi-disciplinary group practices including Aboriginal Community health centres.

Participants in the APCC program included GPs, practice nurses and practice managers.

These staff were encouraged and supported to introduce small, manageable changes in specific areas during the activity periods between workshops. By 2014 over 1800 practices across Australia had participated in at least one program wave (approximately 20% of registered primary care practices in the country) [9]. A before-after evaluation of seven APCC waves involving 743 practices and 150,000 patients over the period 2004–2009 showed improvements in four of the five program areas, with the exception of health care access [5].

For over eight years IF provided QI support to practices directly, however in 2012 the funding contract changed and required them to provide support to government-funded, meso-tier primary health care organisations (PHCO) (previously called Medicare Locals and now called Primary Health Networks (PHN)) who would in turn provide direct support to practices. A review of the program by Knight et al. in 2014 suggested that further institutionalising the QIC from within a PHCO had potential to improve program utilisation, sustainability and spread [4]. The new PHN contracts in 2016 provide an opportunity to capitalise on this potential with the specific inclusion of quality improvement activity to assist in increasing and improving the efficiency and effectiveness of medical services for patients [12]. From 2019 PHN’s will be required to support general practices undertake QI programs and to offer assistance in oversight and management of practice data. General Practices will be offered an incentive payment to share their data with an external organisation such as a PHN.

In this paper we report on a qualitative study that sought to identify enablers and barriers to implementation of sustainable QI programs in the primary care setting.

## Methods

### Participants

Participants for this study were purposively sampled from APCC program staff and practice team members who had participated in QI from around Australia, some of whom were known to the primary researcher through the APCC program. Interviewees were sequentially selected to provide multiple perspectives, focussing on program governance, operational aspects and front-line clinician experience with varying levels of prior knowledge about Quality Improvement. Three of the interviewees were employees of a PHCO and all the clinicians had personal experience of interacting with local PHCO’s. The background and focus of the study were explained to participants prior to commencing the interviews.

### Data collection

Semi-structured telephone interviews were conducted from December 2014–June 2015, ranging from 45 to 60 min in duration. Topic questions were sent via email prior to the interview (see Additional file 1). Participants were invited to reflect on their personal experience of the APCC program, and perceived enablers and barriers to completing the program and factors influencing ongoing participation. Participants were also asked to consider what level of support had been required from their local PHCO to assist practices and local regions to achieve sustainable QI practice. Interviews were recorded, professionally transcribed and supplemented with notes taken by the interviewer.

### Analysis

The primary researcher (CH) reviewed the transcripts, conducted a thematic analysis, and prepared synthesis memos which were discussed with each of the other authors. A phenomenological approach [13] was taken to understand participants’ perceptions, perspectives and understanding of the implementation of the APCC program. An initial analysis of five transcripts was used to guide subsequent selection of interviewees by identifying areas where it was helpful to have more data regarding a particular experience (e.g. to gain an increased understanding of the barriers experienced by practice teams). The primary researcher developed descriptors of the emerging themes using a constant comparative approach with themes emerging during iterative review of the interviews. These descriptors were refined following discussion with the research team. There was evidence for data saturation by repetition of themes after 15 interviews. The resultant themes were synthesized into a conceptual model and presented for feedback at four primary care professional conferences during 2015 (see Additional file 2). Feedback from participants at these presentations (Primary care clinicians and staff from Improvement Foundation) was used to re-format the initial thematic groupings, refine the model and elaborate on the study implications. Interviewees were provided with the opportunity to review the presentation findings and ensure their views were adequately represented.

## Results

Fifteen people were invited via email and all agreed to participate in the study.

Participant characteristics are listed in Table 1.

**Table 1:** Demographic details of participants

	Male	Female	Rural	Urban	GP	Practice Nurse	Practice Manager	Totals
Primary Care	6	3	6	3	7	1	1	9
Primary Health care organisation (PHCO)	1	2	2	1				3
APCC staff	1	2						3
Totals	8	7						15

The identified QI implementation enablers and barriers were grouped into five thematic areas: Leadership, organisational culture, funding, data and clinical systems. These thematic areas are described below.

### Leadership: The value of identifying and supporting change champions

Good leadership by change champions was identified as essential for all three levels of the primary care environment– individual GPs, the practice team and the PHCO itself.

Primary care teams that were most successful at adopting QI culture were able to identify the presence of a change champion, generally described as a GP leader who had the ability to enable change within their group practice and a willingness to adopt and model innovative clinical practices. Several interviewees discussed the role of the PHCOs in facilitating both practice managers and practice nurses, in addition to GPs, to be emergent leaders and change champions for QI within their work environment. These staff were often less visible compared to GPs but identified as “power brokers” for facilitating system redesign and change of clinical practice.

A critical issue identified by all interviewees was the extent to which individual GPs and practice teams were willing to adopt new ideas or change current practice and the role of leadership in effecting change. Several interviewees adopted the language of Rogers’ Diffusion of Innovations theory [14] to describe clinicians and practice teams willingness to change within the APCC program i.e. early adopters, early majority, late majority and laggards. GPs who were considered early adopters in the program, were identified as playing a key role in developing, sharing and adapting change ideas. Change champions often emerged during the APCC process from amongst early adopters e.g. after experiencing small changes within their own practice or whilst participating in high level peer discussions and sharing success stories at face to face meetings with colleagues.

Conversely, GPs who were more resistant to adoption of change ideas, the late majority, had the potential to block QI within a practice team. It was noted that sometimes there was greater variation in clinical practice within a team than between different practices and that this may be better addressed via the shared stories of “external” change champions and clinical leaders.

*“So the majority of the practices who sit in that middle part of the change innovation bell curve (the early and late majority) seem to be more willing to start to engage by using the example of ideas shared by other practices who are early adopters or change agents.” 8PC*

It was also suggested by some interviewees that GPs who were most resistant to adopting changes (laggards) were “not worth pursuing” due to the increased effort required for very little tangible change in the short term.

### Organisational culture: Empowering primary care practices to embody a culture of quality improvement

Organisational culture refers to the shared values and beliefs of a primary care team which governs how the people within the team behave. Organisational culture was seen as having a strong influence on the way in which Quality Improvement (QI) ideology was adopted and embraced within the primary health care setting. The ability to consider and adopt change ideas was a key feature of practices that were described as being successful in QI methodology. Interviewees also spoke about “capacity” for QI as being a driver within the primary care arena. This capacity was influenced by GPs’ motivation to participate, their knowledge of QI and their ability to implement it within the practice setting, as well as access to staff with the skills required to do improvement work. There was strong recognition from all interviewees regarding the need for PHCOs to facilitate QI work within the practice setting. PHCOs that had pre-existing strong relationships with practice teams, and had previously provided hands-on support, practice coaching, professional education and peer to peer mentoring, were best placed to fulfil this facilitation role. Interviewees considered PHCO staff needed to play the role of coach and educator when facilitating QI work. When these roles were performed well, interviewees perceived this to result in better teamwork, more reliable data collection, and increased use of PDSA cycles, improved guideline use, development of change champions and sharing of success stories.

*“Practice support has got to be the main job of the Medicare Local, and they need to become deeply knowledgeable about the how to’s….the model of support is more like that of a coach rather than a trainer.” 7PC*

Interviewees noted the critical need for PHCOs to establish trusted relationships with practice staff. This enabled both easier access by the PHCO staff to practices and a locally tailored approach to supporting QI initiatives that recognised the varied abilities of practice teams to engage in the program. These relationships were considered to vary greatly across the country, depending on both individual PHCOs and the staff. It was noted that government changes to the structure of PHCOs in Australia over the past five years had also resulted in changes to prioritisation of QI and their capacity to support General Practice. Interviewees noted a high level of dissatisfaction regarding the decreased prioritisation for general practice support from the former Medicare Locals, and opportunities to redress this with the formation of new Primary Health Networks.

A benefit of the collaborative nature of the QI process, as experienced through the APCC, was the ability for primary care teams to share ideas and solutions for common problems, which helped to build a culture of “Improvement” within practice teams.

Interviewees described how participants in the QI process valued the conversations among clinical peers about clinical decision making. This included the value of working as a team member rather than working autonomously, whether that be in a group practice or as a solo clinical practitioner surrounded by non-clinical team members. Many GPs commented that they were working in systems designed to maintain individual clinician autonomy and this served as a barrier to a more systematised team-based approach.

*“I think the biggest barrier really is practice culture, if the practice is really a building where a lot of independent GPs see a lot of patients, they’re not really keen to work as a team or review practice data – this isn’t really what they’re on about in terms of managing a population. So I think one of the barriers is actually philosophical.” 10 PC*

For clinicians within larger practices this ‘philosophical’ barrier was often overcome by peers praising the value of working as a part of a team with shared responsibility for patient care and a reduced feeling of being overwhelmed by the workload, resulting in easier implementation of team based systems.

### Financial incentives: The role of funding incentives to generate change

GPs in Australia are primarily remunerated under Medicare - a public, universal insurance scheme based on a scheduled fee for face-to-face consultations and procedures. General Practices that have met accreditation standards are also able to access additional funding called “Practice Incentive Payments” (PIP) which are not directed to specific GPs and are linked to specific Government targets such as immunisation and adoption of digital systems. Over sixteen years Medicare has also introduced specific service items that encourage planned and preventive health care. However, there is currently no reimbursement for specific quality improvement related work. Participants all commented about the barrier of current funding mechanisms for promoting QI activities.

*“I think the experience has clearly shown that GPs will do what brings in money, because we are small business people and we do need to fund what we’re doing.” 13 PC*

This poses challenges to engagement with QI work because “quality” may not equate to increased income, and could even lead to decreased revenue under the current funding model.

Participants commented on the role of PHCOs in assisting practices develop innovative systems for service delivery that could lead to both improved financial benefits and quality health outcomes by aligning service incentive payments with systematised quality care planning. They commented that this helped to address reluctance to participate in work that was perceived to be of low value from a business revenue perspective. There was general consensus that lack of financial incentives could also be partially addressed if PHCO staff provided hands on support within practices to conduct IT related work such as data extraction and analysis.

### Data: The transformational value of good clinical data systems

All interviewees emphasised the crucial role that data and IT systems played in participation and successful implementation of QI by general practice teams. By “data” interviewees were referring to coded clinical information within electronic health records amenable to data extraction to generate practice and/or GP specific reports. Interviewees all identified that the quality of data and the ability to provide regular accurate reports about practice populations was key to enabling QI work. For example, if a practice used software that was compatible with the data extraction tools then they were able to easily adopt QI ideas to improve their data quality. In contrast, Practices using software that was not compatible found it too challenging to try and create their monthly data reports.

Insufficient technical support and expertise within practices around data and IT systems were identified as a significant barrier in the uptake of QI work. All interviewees talked about the frustrations experienced by practice teams over difficulties of “data cleansing”, data extraction, generation of reports, interconnectivity of IT systems and establishing uniform coding systems amongst the clinicians. Clinician interviewees emphasised the need for hands on “doing” support from PHCO staff such as assisting in data downloads and generating useful reports. APCC staff also emphasised the PHCOs role as a mentor, imparting knowledge and training about systems and data.

All interviewees discussed the key role that PHCOs could play in assisting practice teams to understand the power of having accurate medical records and improved data quality. The APCC program required practices to generate monthly reports against specific clinical measures. Interviewees reported that on average it took practices six months to “clean up” data before they could use the reports to find possible gaps in care. It was felt that most GPs lacked training to fully appreciate the benefits of measureable data and consequently needed support in learning how to record and utilise clinical data in meaningful ways.

*“You need to engage people in constantly measuring their outcomes, understanding the gap and saying ‘how do we then bridge the gap?’. Most people need leadership down that path…..a clear aim and some structure to take participants on a long journey because it’s not as simple as just turning on or off a switch.” 6 PHN*

“High performing” practices demonstrated structured approaches to IT and data management. They had systems for uniform data entry and coding and documented procedures for systems of care such as Diabetes annual cycle of care. PHCOs were seen as a potential conduit for sharing these systems amongst other practices.

### Structured clinical systems: Improving health outcomes through organised frameworks of care

The final theme describes the need for GPs, practice teams and PHCOs to use structured systems as an overarching framework to enable implementation of QI methodology and achieve desired outcomes. Thus the work of change champions, adoption of QI culture, financial drivers and data driven improvements were considered to be enhanced by well organised clinical systems.

Interviewees commented that successful practices designed systems that were streamlined (automated if possible) and easy to adopt. Clinicians (GPs and practice nurses) would not adopt changes that took more time and were difficult to fit into their consultation routine no matter how important they may have appeared clinically.

*“It’s got to make it easier to do the right thing. So, yes, benefitting patients is certainly an important part of that, but actually, if it takes me three times as long to do that same task, it’s not going to happen, so it’s got to make it easier for the clinician to do the task as well.” 8 PC*

Sharing of success stories via webinars or at workshops from other practice teams facilitated the development of user friendly clinical systems.

Interviewees considered that PHCOs would be well positioned to establish local QI networks or forums where clinicians were provided with an opportunity to discuss high level evidence, current best practice and practical ways to achieve better health outcomes both at an individual and practice level through system redesign. There was general consensus that a standout benefit of the APCC program had been the access to a network of likeminded peers that facilitated discussion around the design of systems for improving health outcomes in the primary care setting.

Interviewees noted that ensuring all team members were engaged in the adoption of changes in systems also assisted in minimising the problem of loss of ‘corporate memory’ due to staff turnover. This was particularly identified as a barrier when there had only been one or two staff members assigned to the role of overseeing QI projects and implementation. Practice teams who achieved higher success in the APCC program outcomes were noted to have developed communication systems inclusive of all team members and espoused a philosophy of teamwork and systematised care.

## Discussion

The APCC program has provided a rich context for identification and analysis of enablers and barriers for QI in the Australian setting. Participants of the program provided useful insights into possible future implementation strategies.

Health systems internationally are investing in primary care meso-tier organisation to reduce fragmentation and improve system performance. Whilst there is substantial variation in the specific roles they play in the system, ranging from commissioning (Clinical Commissioning Groups in England [15]), financial accountability and provision of financial incentives (Accountable Care Organisations in United States of America [16]), direct service provision (Primary Health Organisations in New Zealand [17]), engagement in quality improvement activities is a common element and therefore the themes found in this study are likely to be relevant in an international context.

Primary Health care is a complex environment that benefits from structured systems of care to assist the adoption of best practice. The privatised model of Australian general practice acts as financial and philosophical barrier to widespread adoption of QI programs. Primary Health Networks (PHN) have an opportunity to assist in countering these barriers and implement solutions that are tailored to local health care needs.

Studies regarding the role of meso-level organisations in primary health care in UK, Canada and New Zealand suggest that such organisations can play an important role in facilitating a more integrated health system and promoting peer collaboration with resultant improvements in efficiency and quality of care [18–21]. The impact of PHCO’s such as PHNs could be optimised by emphasising relationship building between the external facilitators and general practices. Trusted relationships have been demonstrated to play a key role in assisting adoption of evidence based improvements in the healthcare environment [22, 23]. Ideally these trusted relationships will be established at both the individual and organisational level.

The five study themes identified in this study (leadership, organisational culture, funding, data and clinical systems) are closely aligned with four out of ten building blocks of high performing primary care practices described in 2014 by Bodenheimer et al. [24]. The 10 Building Blocks framework describes leadership, data driven improvement, empanelment (patient registration) and team based care as the foundation for implementation of a model for innovative thinking, improvement and primary care transformation. The challenge for the primary care setting both internationally and in Australia will be to identify and mentor local clinical leaders and change champions to facilitate the adoption of QI. The role of PHCO’s in overseeing the process as well as demonstrating organisational culture in keeping with QI will be critical. This resonates with the findings of Kaplan et al. who reviewed 47 articles regarding QI in healthcare and linked success of QIC programs to high level leadership, organisational culture, data infrastructure, clinician involvement and the number of years involved in QI programs [8]. Nicholson et al. in 2013 discussed the role of meso-level primary care organisations in realising integrated health system reforms and identified ten key governance elements as key for PHCOs success, including measurement and data for quality improvement, incentives and professional education [25]. This aligns with our finding that PHCOs be tasked with a substantive facilitator role in QI implementation. In practical terms this requires PHCO staff effectively engaging and supporting individual GPs and practice teams in QI processes. Specific suggestions for such processes are outlined in Table 2.

**Table 2:** Suggested process through which PHCOs can support QI Implementation in general practice

•Training: Practice Coaching, CPD events, webinars, small groups	**ᅟ**
• Educate: Improvement theory, clinical microsystems, PDSA, evidence based guidelines	**ᅟ**
• Practice support: Data management / Point of care decision tools, Strong relationships with the General Practices, IT/IM, accreditation support, upskill practices to be ready to adopt QI work	**ᅟ**
• Modelling: Sharing of stories and successes by early adopters / QI Networking of General Practices	**ᅟ**
• Leadership Identification: Support leadership training across the region	**ᅟ**
• Incentivisation: Showcasing financial framework / accessing innovative funding	**ᅟ**

The process of GP clinical decision making that assists the adoption of evidence based guidelines is complex. Gabbay and le May coined the term “mindlines” to describe the GP process of internalising tacit guidelines, informed by professional reading and social interactions (opinion leaders, patients, colleagues), resulting in a socially constructed response to guidelines rather than a rigid protocol [26]. Peer networks can assist GPs in the active formulation of these “mindlines”. A PHCO-facilitated network could provide an evidence-based, social and professional platform for these interactions and establish trust between both the PHCO and individual practice teams and GPs. An example would be a group of GPs meeting to network about QI topics, once a month at a time and venue of their choice, facilitated by the PHCO.

PHCOs may also assist GPs in the philosophical adoption of system change within their workplace. For instance, the change in emphasis to systematised team based care has not been widely adopted in the Australian general practice setting and this is likely to be related to a perceived loss of individual GP clinician’s autonomy such as described by Hall in 2009 regarding professional cultures as barriers to interprofessional teamwork [27]. PHCOs can share stories of change from other local general practices that illustrate the benefits of team based care such as improved time management and decreased stress via the peer networks.

To engage individual practitioners long term, it is important to recognise the role of both practice structures and financial incentives. Under the current Australian payment model, lack of direct financial incentives for quality improvement is a significant barrier for many GPs and practice managers. This can affect practice teams’ ability to quarantine sufficient time and resources to implement QI programs. PHCOs are well-positioned to assist practice teams through provision of staff and IT tools. Long term engagement in QI programs will also require modification to the fee-for-service funding models. The Australian government is currently trialling a new model of payment for patients with chronic and complex disease where practices will receive a bundled payment to manage patients whom agree to be enrolled in a “healthcare home” [28] .This is a substantive health reform that has potential to shift primary care remuneration to a more outcomes focussed payment model, and there may be important opportunities for PHCOs to engage clinicians and practice teams in making this transition.

We note the following study limitations. Firstly the interviewees did not include those from general practices with no contact with APCC. While this sampling aligned with the study goals of eliciting lessons from participants’ experience of the APCC, their views may reflect a potential bias toward philosophical alignment with QI. Further study among non APCC participants may identify additional barriers to QI implementation. We also note that the first author was known to all the interviewees due to her involvement in APCC from 2008 until 2012 as a participant and clinical lead. While this allowed potential for some degree of social desirability bias, it was apparent from the interviews that the study participants felt comfortable to report and discuss both positive and negative experiences of the APCC program.

## Conclusion

PHCOs such as the Australian PHNs are well poised to facilitate transformation of primary care through a range of mechanisms identified in this study. However, the challenges to achieving this in the current policy environment are important to recognise and should not be minimised. In developing QI programs and policies, such organisations ought to invest their efforts in: (1) identifying and mentoring local leaders; (2) fostering QI culture via development of local peer networks; (3) developing and advocating for alternative funding models to support and incentivise these activities; (4) investing in data and audit tool infrastructure; and (5) facilitation of systems implementation within primary care practices. If these opportunities are maximised the PHCOs will be well positioned to make a major contribution to improved delivery of health outcomes in the primary care arena.

## Additional files

Additional file 1: Interview Guide – questions sent to all interviewees prior to interview and used as the basis for each interview. (DOCX 13 kb)

Additional file 2: Feedback regarding research findings - Presentations of the findings where feedback was sought from a wider setting. (DOCX 13 kb)

## Abbreviations

APCC: Australian Primary Care Collaborative
GP: General Practitioner
IF: Improvement Foundation
PDSA: Plan, Do, Study, Act
PHCO: Primary health care Organisation
PHN: Primary Health Network
QI: Quality Improvement
QIC: Quality improvement collaborative
