# Supplementary material for: Informing implementation of quality improvement in Australian primary care
Source: BMC Health Serv Res. 2018 Apr 16;18:287. doi: 10.1186/s12913-018-3099-5 (PMC5903003; doi:10.1186/s12913-018-3099-5)
Supplement: Supplementary file 1 — Interview Guide – questions sent to all interviewees prior to interview and used as the basis for each interview. (DOCX 13 kb) [file 12913_2018_3099_MOESM1_ESM.docx]

Additional Files 1

**Interview Guide**

What are Qualities of practices - Predictors of success that are inherent within practices?

What is it in a wave that makes it successful – what particular inputs are provided to enhance success at the practice level?

What are the barriers to success or completion in a wave?

What do you think would achieve sustainable changes in practices?
