# Supplementary material for: Informing implementation of quality improvement in Australian primary care
Source: BMC Health Serv Res. 2018 Apr 16;18:287. doi: 10.1186/s12913-018-3099-5 (PMC5903003; doi:10.1186/s12913-018-3099-5)
Supplement: Supplementary file 2 — Feedback regarding research findings - Presentations of the findings where feedback was sought from a wider setting. (DOCX 13 kb) [file 12913_2018_3099_MOESM2_ESM.docx]

Additional File 2

**Feedback regarding research findings:**

**Presentations of the findings where feedback was sought from a wider setting**

1. Informing Quality Improvement (QI) work in the Primary care setting and

Implementation of a program to support quality improvement activities through

local GP organisations.

Hespe,C, Rychetnik,L, Peiris,D, Harris,M

Poster and Presentation, Primary Health Care Research Conference, Canberra, July

2015

1. Informing Quality Improvement (QI) implementation in the Primary care setting.

Hespe,C, Rychetnik,L, Peiris,D, Harris,M

Presentation, GP15, Annual Scientific meeting, Melbourne, September 2015

1. Informing Quality Improvement (QI) Work in the Primary Care Setting and

Implementation of a program to Support Quality Improvement Activities Through

Local GP Organisations.

Hespe,C, Rychetnik,L, Peiris,D, Harris,M

Presentation, North American Primary Care Research Group (NAPCRG) Annual

Meeting, Cancun, Mexico, October 2015

1. Informing Quality Improvement (QI) Work in the Primary Care Setting.

Hespe,C, Rychetnik,L, Peiris,D, Harris,M

Presentation, Improving Healthcare International Convention 2015, Melbourne, November 2015
